# Hydrogen-rich saline ameliorates hippocampal neuron apoptosis through up-regulating the expression of cystathionine β-synthase (CBS) after cerebral ischemia- reperfusion in rats

**DOI:** 10.22038/ijbms.2020.41751.9857

**Published:** 2020-04

**Authors:** Hai-ming Cong, Qiu-ping Gao, Guo-qiang Song, Ying-xin Ye, Xiao-li Li, Lian-shuang Zhang, Xi-feng Wang

**Affiliations:** 1 Department of Rehabilitation Medicine, Weihai Central Hospital, Wendeng 264400, China; 2 Department of Emergency, Ji Nan Zhang Qiu District Hospital of Traditional Chinese Medicine, Zhang Qiu 250200, China; 3 Department of Neurosurgery, Ji Nan Zhang Qiu District Hospital of Traditional Chinese Medicine, Zhang Qiu 250200, China; 4 Department of Neurology, Ji Nan Zhang Qiu District Hospital of TraditionalChinese Medicine, Zhang Qiu 250200, China; 5 Department of Critical Care Medicine, Yu Huang Ding Hospital, Qingdao University, Yantai, P. R. China; 6 Department of Histology and Embryology, Binzhou Medical University, Yantai, P. R. China

**Keywords:** Cerebral ischemia – reperfusion, Cystathionine β-synthase, Hippocampus, Hydrogen, Rats

## Abstract

**Objective(s):**

This study aimed to evaluate the potential role of hydrogen in rats after cerebral ischemic/reperfusion (I/R) injury.

**Materials and Methods:**

The experimental samples were composed of sham group, model group of rats that received middle cerebral artery occlusion (MCAO) for 2 hr followed by reperfusion for 24 hr, and the hydrogen saline group treated by hydro¬gen-rich saline (1 ml/kg) after MCAO. Hydrogen sulfide (H2S), S100-βprotein (S100-β), and neuron-specific enolase (NSE) levels were measured; the levels of malondialdehyde (MDA), reactive oxygen species (ROS), and superoxide dismutase (SOD) were detected; the histologic structure and apoptotic cells of hippocampus were observed; the expressions of cystathionine β-synthase (CBS), nuclear factor erythroid 2-related factor 2 (Nrf2), and hemeoxygenase-1 (HO-1) were measured. Statistical analyses were performed using one-way analysis of variance (ANOVA) followed by Fisher’s least significant difference (LSD) test.

**Results:**

Our results showed that hydrogen up-regulated H2S levels via promoting the expression of CBS in the hippocampus, and its treatment alleviated oxidative stress via activating the expression of Nrf2 and HO-1, and then cell apoptosis reduced, furthermore, brain function improved by down-regulating the levels of S100-βand NSE.

**Conclusion:**

This study showed that hydrogen-rich saline ameliorates cell injury through up-regulating the expression of CBS in the hippocampus after cerebral ischemia reperfusion (I/R) in rats, this provides new experimental evidence for the treatment of stroke with hydrogen saline.

## Introduction

It is known that ischemic stroke characterized by the sudden loss of blood circulation is a major type of stroke (1) and has become the second leading cause of death globally (2, 3). Moreover, its high recurrence rate has economic burdens for the society. Studies have confirmed that cerebral ischemia has a complicated pathology closely related to oxidative stress (4-7). Accumulating evidence demonstrated that the main cause of neuron damage is not ischemia itself but the overproduction of reactive oxygen species (ROS) which attacks cells, resulting in ischemia/reperfusion (I/R) injury and neuronal cell death (8-10). Thus, a better understanding of the molecular and cellular mechanisms underlying oxidative stress injury after I/R may provide novel treatment for ischemic stroke. It has generated considerable interest in developing antioxidant therapies to combat ischemia-induced damage due to the close relationship between cerebral ischemia and oxidative stress (11). 

Hydrogen sulfide (H_2_S), known as a toxic gas in nature (12) with an odorous smell, is synthesized from L-cysteine by enzymes such as cystathionine γ-lyase (CSE), cystathionine β-synthase (CBS), and mercaptopyruvate sulfurtransferase (3MST) (13). CBS is predominantly responsible for the production of H_2_S in the central nervous system (14, 15), recent studies have shown that small amounts of H_2_S are produced in the brain (14), and it has been proven to be an endogenous factor that regulates cellular function (16). Previous research has investigated that H_2_S has a protective effect against cerebral injury in rodent models (17). So, up-regulating the expression of CBS to promote H_2_S synthesis may be a therapeutic strategy for stroke.

Hydrogen is a gas that can be used for diving (18). However, studies have confirmed its anti-oxidative capabilities in animal models since Ohsawa *et al*. reported that hydrogen inhalation could protect the brain against cerebral I/R injury (19). In our previous researches, we also found that hydrogen has a neuroprotective effect in ischemia reperfusion rats (20, 21). The novelty of this study is investigating whether hydrogen protected the brain against I/R injury through up-regulating the expression of CBS and changing the levels of neurological function indices such as neuron specific enolase (NSE) and S100-βprotein (S100-β) in the hippocampus and its related mechanism.

## Materials and Methods

***Preparation of hydrogen-rich saline***

The hydrogen-rich saline was purchased from Second Military Medical University (Shanghai, China) and stored under atmospheric pressure at 4 ^°^C in an aluminum bag. Hydrogen-rich saline was freshly prepared within one week to ensure a constant concentration no less than 0.6 mmol/l (22).

***Experiment design***

In this study, 36 adult male Sprague-Dawley Rats (weighing 280–320 g) were obtained from the Experimental Animal Center of Shandong University (Jinan, China). The experiment protocols were approved by the Ethics Committee of Bin Zhou Medical University and performed in accordance with the guidelines of National Institutes of Health Guide for Laboratory Animals.

After one week acclimation, the rats were divided into three groups randomly, 12 rats in each group. The animals were anesthetized with 3.5% chloral hydrate solution (1 ml/100 g, IP). Middle cerebral artery occlusion (MCAO) was induced as our previous research described (21). In brief, the left common carotid artery (CCA) and the external carotid artery (ECA) were exposed, and then, a 3-0 surgical monofilament nylon suture was inserted from the external carotid artery into the internal carotid artery (ICA) carefully and was advanced in a forward manner to occlude the origin of the left middle cerebral artery (MCA) until a light resistance was felt (18–20 mm from the CCA bifurcation). After 2 hr of MCAO, the nylon suture was withdrawn, followed by 46 hr of reperfusion. Twelve rats were used as sham group (suture only after exposure of carotid artery), 12 rats were used as I/R group after MCAO, and the I/R + hydrogen group comprised 12 MCAO rats treated with hydrogen-rich saline (1 ml/kg) after the beginning of reperfusion. After 24 hr, all rats were euthanized with chloral hydrate (7%, 5 ml/kg). The blood and brain tissues from each animal were collected for analysis. 

***Measurement of S100-βand neuron-specific enolase (NSE) levels***

The blood samples were allowed to clot and then centrifuged at 3,000 rpm for 10 min, sera were separated and used to determine S100-β protein and NSE by Enzyme-Linked Immunosorbent Assay (ELISA) (Yuchen, Shanghai) according to the manufacturer’s protocol.

***Measurement of H***_2_ ***S levels***

The biosynthesis of H_2_S in the brain was measured as described previously (24). The optical absorbance was measured at 655 nm with a microplate reader (iMark; Bio-Rad). The H_2_S concentration of each sample was calculated.

***Measurement of ROS, malondialdehyde (MDA) and superoxide dismutase (SOD)***

ROS and SOD are the indicators of oxidative stress. The homogenates of brain tissue were centrifuged at 3,000 rpm for 20 min at 4 ^°^C. The supernatant was separated and the activity of ROS and SOD were determined using a detection kit (Jiancheng, China) as manual protocol. Optical density was determined using a spectrometer, both at 550 nm.

The concentration of MDA as a marker of lipid peroxidation, was measured using a detection kit (Jiancheng, China) following the manufacturer’s protocol. Optical density was determined by a spectrometer at 532 nm.

***Histopathological examination***

Isolated ischemic cerebral tissues were fixed with 10% methanol, embedded with paraffin, tissues were sectioned at a thickness of 5 μm, stained with hematoxylin and eosin (HE), and observed under a light microscope (Olym-pusX71-F22PH, Japan) at 400 magnification. The total and injured neurons were counted in 12 different fields of microscope per sample, six samples in each group were counted.

***Immunohistochemical staining***

Immunohistochemical staining method refers to our previous studies (21).The ischemic cerebral tissues were fixed in 10% formalin, embedded in paraffin, and cut into 5-μm thick sections, stained with a CBS antibody (diluted 1:500, Cell Signaling Technology (CST), USA), followed by a second IgG antibody. Immunostaining was performed with diaminobenzidine (DAB).The DAB staining density was assessed with a microscopic image analysis system (GX51, Olympus, Japan).

***TdT-mediated dUTP nick end labeling (TUNEL)***

The ischemic cerebral tissue was fixed in 10% formalin, embedded in paraffin, and sectioned at a thickness of 5 μm; TUNEL staining was performed with an *in situ* cell death detection kit (Jiancheng, China) according to the manufacturer’s protocol. 

First, the sections were rinsed with PBS and were treated with 1% Triton X-100 for 3 min. Terminal deoxynucleotidyl transferase (TdT) was used to catalyze the addition of biotinconjugated dUTP to the 3′-OH ends of the DNA fragments subsequently. Streptavidin-HRP solution was added and reacted at 37 °C for 30 min. Finally, the slides were placed in DAB for 3 min and stained with Hematoxylin Harris. These analyses were performed at 100× magnification under a light microscope in 12 different fields using computer-aided software (Olympus X71-F22PH, Japan). The apoptosis cells were quantified using computer assisted image analysis (Leica LAS Image Analysis V4.0).

***Western blotting***

The protein was extracted using an extraction kit (Beyotime Biotechnology, china). Equal amounts of lysate proteins (20 μg) were loaded onto SDS-polyacrylamide gels and electrophoretically transferred to polyvinylidene fluoride (PVDF) membranes (Millipore, USA). After blocking with 5% nonfat milk in TBS and Tween 20 (TBST) for 1 hr, the PVDF membrane was incubated with β-actin (1:2000, CST, USA), Nrf-2 (1:800, CST, USA), HO-1(1:800, CST, USA) overnight at 4 ^°^C. Then, membranes were washed three times with TBST and incubated with IgG secondary antibody (1:5000, Beyotime Biotechnology, China) for 1 hr at room temperature. After washing with TBST, the antibody-bound proteins were detected with the ECL chemiluminescence reagent (Beyotime Biotechnology, china). Protein levels were calculated relative to that of β-actin. The images were analyzed using the software package (Bio-Rad Laboratories Inc, Hercules, CA, USA).

***Statistical analysis***

When data were normally distributed, they were analyzed using SPSS 21.0 and are expressed as mean ±standard error (SEM). Statistical analyses were performed using one-way analysis of variance (ANOVA) followed by Fisher’s least significant difference (LSD) test, and a value of *P*<0.05 was considered statistically significant.

## Results

***Effect of hydrogen on H***_2_ ***S levels***

**Figure 1:**
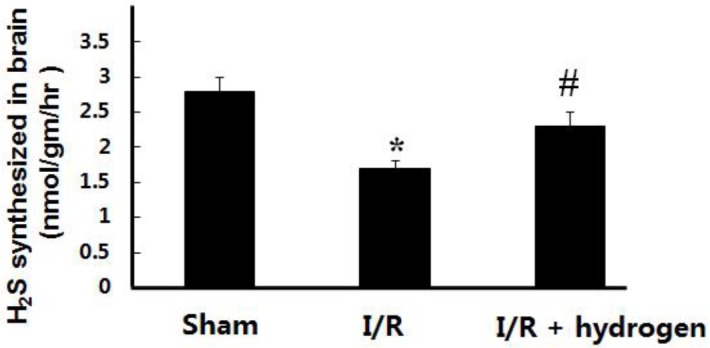
Effects of hydrogen on hydrogen sulfide levels in middle cerebral artery occlusion rats (mean±SEM). * indicates significant difference compared with sham group (*P*<0.05), # indicates significant difference compared with I/R group (*P*<0.05). I/R: ischemia/reperfusion

The levels of H_2_S in the brain tissue decreased in the I/R group compared with the sham group (*P*<0.05), but hydrogen increased the endogenous H_2_S levels in the brain compared to the I/R group (*P*<0.05) (Figure 1). 

***Effect of hydrogen on S100-***β***and NSE levels***

**Figure 2:**
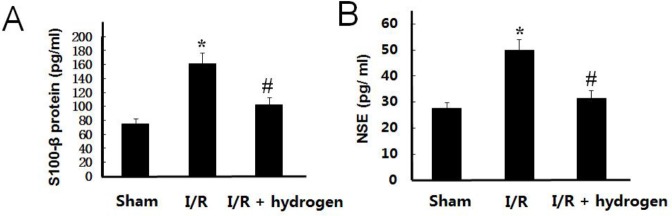
Effects of hydrogen on S100-βprotein and neuron-specific enolase levels in middle cerebral artery occlusion rats (mean±SEM). S100-β protein level in brain (A), neuron-specific enolase level in brain (B). * indicates significant difference compared with sham group (*P*<0.05), # indicates significant difference compared with I/R group (*P*<0.05). I/R: ischemia/reperfusion

The levels of S100-β and NSE have similar trends, these increased in the I/R group compared with those of the sham group (*P*<0.05), but hydrogen treatment decreased their levels in the brain compared to the I/R group (*P*<0.05) (Figure 2). It indicated that hydrogen played a protective role against ischemic injury.

***Changes of histopathological structure***

**Figure 3:**
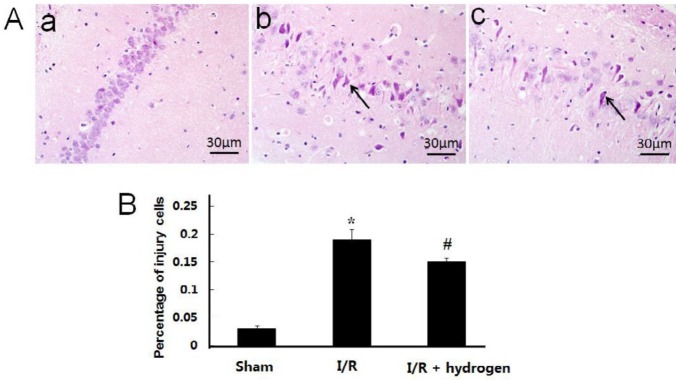
Histopathological observation of the hippocampus in middle cerebral artery occlusion rats. Sham group (a), I/R group (b), I/R+hydrogen group (c). Black arrows denote neurons in the hippocampus. HE ×400 (A). Percentage of injury cells in the three groups (B). Scale bar: 30 μm. * indicates significant difference compared with sham group (*P*<0.05), # indicates significant difference compared with I/R group (*P*<0.05). I/R: ischemia/reperfusion

The neurons in the CA1 area of the hippocampus were observed. Normal neurons had round nuclei, but the nuclei of necrotic neurons were irregular, shrinkage, and with deep staining. Figure 3A shows obvious morphological changes in the I/R group in which the body and nuclei of neurons were reduced with shrinkage, and percentage of injured cells detected in the I/R group was significantly higher than that in the sham group (*P*<0.05), but it decreased after hydrogen treatment (*P*<0.05) (Figure 3B). 

***Effect of hydrogen on ROS, MDA, and SOD levels***

**Figure 4:**
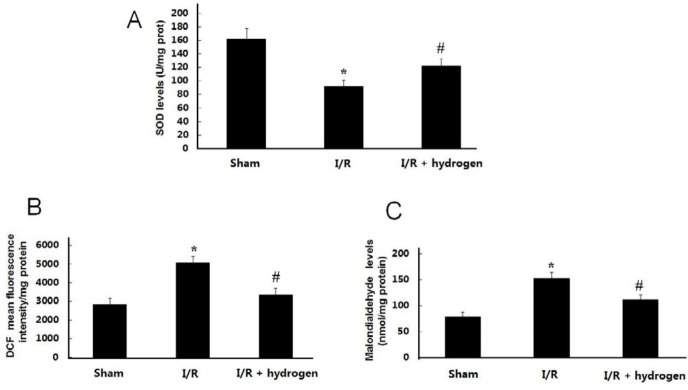
Effects of hydrogen on reactive oxygen species, malondialdehyde and superoxide dismutase levels in middle cerebral artery occlusion rats (mean±SEM). SOD level in the brain (A), ROS level in the brain (B), MDA level in the brain (C). * indicates significant difference compared with sham group (*P*<0.05), # indicates significant difference compared with I/R group (*P*<0.05). I/R: ischemia/reperfusion; SOD: superoxide dismutase ; ROS: reactive oxygen species

As shown in Figures 4 B and C, ROS and MDA levels were much higher in the I/R group than those in the sham group (*P*<0.05). Treatments with hydrogen decreased ROS and MDA levels compared with the I/R group (*P*<0.05). On the contrary, the levels of SOD in the brain tissue decreased in the I/R group compared with the sham group (*P*<0.05), but hydrogen increased the endogenous H_2_S levels in the brain compared with the I/R group (*P*<0.05) (Figure 4A). 

***Changes of apoptosis***

**Figure 5:**
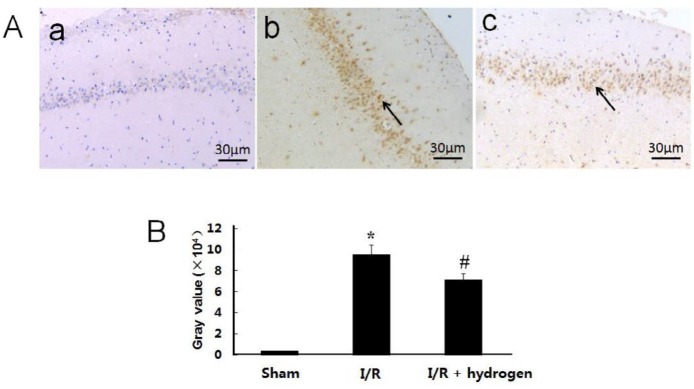
Effects of hydrogen on expressions of TdT-mediated dUTP nick end labeling positive cells in middle cerebral artery occlusion rats. Sham group (a), I/R group (b), I/R+hydrogen group (c). Black arrows denote TUNEL positive neurons in the hippocampus (A). Gray values of the TUNEL positive cells in the three groups (B). Scale bar: 30 μm. * indicates significant difference compared with sham group (*P*<0.05), # indicates significant difference compared with I/R group (*P*<0.05). I/R: ischemia/reperfusion

The TUNEL staining is shown in Figure 5A, the gray value of positive TUNEL cells found in the I/R group was significantly higher than that of the sham group (*P*<0.05), but it decreased after hydrogen treatment (*P*<0.05) (Figure 5B).

***Changes of CBS expression***

**Figure 6:**
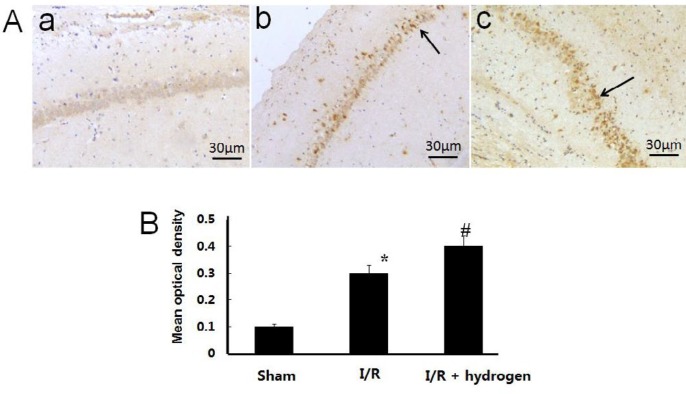
Effects of hydrogen on the expression of cystathionine β-synthase in middle cerebral artery occlusion rats. Sham group (a), I/R group (b), I/R+hydrogen group (c). Black arrows denote cystathionine β-synthase positive neurons in the hippocampus (A). Gray values of cystathionine β-synthase positive cells in the three groups (B). Scale bar: 30 μm. * indicates significant difference compared with sham group (*P*<0.05), # indicates significant difference compared with I/R group (*P*<0.05). I/R: ischemia/reperfusion

The expression of CBS showed brown staining in cells (Figure 6 A). Its mean density in I/R group increased compared with the sham group (*P*<0.05). Moreover, it increased significantly higher in the hydrogen treatment group compared with I/R group (*P*<0.05) (Figure 6 B). It indicated that hydrogen up-regulated the CBS expression in ischemic hippocampus.

***Changes of Nrf-2 and HO-1 expressions***

**Figure 7:**
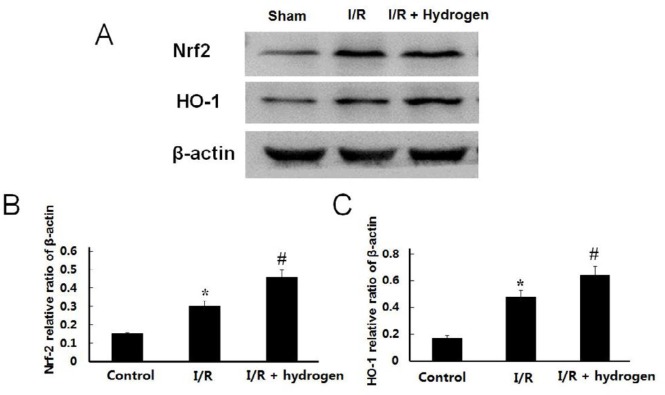
Effects of hydrogen on protein expression in middle cerebral artery occlusion rats. Expressions of Nrf-2 and HO-1in each group (A). Relative expressions of Nrf-2 (B) and HO-1 (C) in each group were measured. Data are reported as means±SE. * indicates significant difference compared with sham group (*P*<0.05), # indicates significant difference compared with I/R group (*P*<0.05). I/R: ischemia/reperfusion; Nrt2: nuclear factor erythroid 2-related factor 2; HO-1: hemeoxygenase-1

The expression levels of Nrf-2 and HO-1 increased in the I/R group compared with the sham group (*P*<0.05), and the hydrogen treatment further up-regulated the protein levels of those compared to the I/R group (*P*<0.05) (Figure 7). It indicated that endogenous expression of Nrf-2 and HO-1 were activated after I/R, and hydrogen could further up-regulate the expressions of those.

## Discussion

In the present study, we investigated the protective role of hydrogen in rats after I/R injury through up-regulation of H_2_S levels, it was confirmed by H_2_S assessment and CBS expression in the hippocampus. Meanwhile, the treatment of hydrogen reduced oxidative stress and down-regulated the percentage of apoptotic neurons in ischemic hippocampus, and then injuries of neurons were improved. These results indicated the protective effect of hydrogen on injured neurons. These are consistent with our previous research showing that hydrogen protected the neurons against I/R injury (20, 21). Furthermore, the novelty of this study is that hydrogen may achieve its protective effect by up-regulating the expression of CBS and H_2_S levels in the hippocampus. 

H_2_S is the third gas signal molecule (24), has been recognized to play crucial physiological functions in the central nervous system (25, 26). The results were in accordance with previous reports that the expression of CBS and concentration of H_2_S increased in the hippocampus of rats after brain ischemia (27), it indicated that hydrogen up-regulated the H_2_S levels in ischemic brain, and H_2_S could reduce cerebral I/R injury in the animal model (28-31). To further elucidate the mechanism of hydrogen in alleviating MCAO-induced cerebral ischemic injury, the present study investigated the effects on antioxidants. Oxidative stress is a core pathological component closely related to reperfusion injury accompanied with excessive ROS production (32, 33). Our results demonstrated that induction of I/R leads to elevated levels of ROS and MDA and a decrease of SOD. Increasing studies have shown that treatment with H_2_S could inhibit apoptosis via blocking an ROS activated Ca^2+ ^signaling pathway in hypoxia-induced hippocampal neurons (30) and improved ischemic damage and apoptosis in cerebral ischemia through its antioxidant effects (34-36). As expected, we found that treatment with hydrogen reduced ROS and MDA levels in the ischemic brain of I/R rats, and increased the SOD activity as well as the expression of CBS, nuclear factor erythroid 2-related factor 2 (Nrf2), and hemeoxygenase-1 (HO-1). All these implied that treatment with hydrogen saline significantly suppressed oxidative stress in ischemic brain. Nrf2 is an endogenous cytoprotective factor that activates the transcription of antioxidant stress genes, including HO-1 against oxidative stress (37, 38). The activation of Nrf2/HO-1 antioxidant pathway has been shown to play an important neuroprotective role after ischemia reperfusion-induced brain injury (39-41). The results showed that hydrogen significantly regulated Nrf2/HO-1 levels in the ischemic model.

## Conclusion

The present study demonstrated that hydrogen could protect neurons in the hippocampus against ischemic injury through up-regulation of the CBS expression and activating the Nrf2/HO-1 antioxidant pathway. This provides a new experimental basis for clinical application of hydrogen in the treatment of cerebral ischemic injury. But the specific mechanism of how hydrogen up-regulated CBS expression remains to be further explored, this is the subsequent target for us and other researchers.
